# WT1 peptide vaccine in Montanide in contrast to poly ICLC, is able to induce WT1-specific immune response with TCR clonal enrichment in myeloid leukemia

**DOI:** 10.1186/s40164-018-0093-x

**Published:** 2018-01-11

**Authors:** Hongtao Liu, Yuanyuan Zha, Noura Choudhury, Gregory Malnassy, Noreen Fulton, Margaret Green, Jae-Hyun Park, Yusuke Nakamura, Richard A. Larson, Andres M. Salazar, Olatoyosi Odenike, Thomas F. Gajewski, Wendy Stock

**Affiliations:** 1 Section of Hematology/Oncology, Department of Medicine, University of Chicago Medical Center, 5841 S. Maryland, MC 2115, Chicago, IL 60637-1470 USA; 2 HIM Facility at University of Chicago, University of Chicago Medical Center, Chicago, IL USA; 3 Internal Medicine Residency Program, The University of Chicago Medicine, Chicago, USA; 4 Oncovir, Inc., Washington, DC USA

**Keywords:** WT1, Vaccine, TLR3, AML

## Abstract

**Background:**

The optimal strategy for vaccination to induce CD8^+^ T cell responses against WT1 is not known.

**Methods:**

A pilot randomized study in HLA-A02^+^ patients to receive vaccination with WT1 in Montanide or in poly ICLC, a TLR3 agonist, to explore the novel immune adjuvant was conducted. Seven patients were randomized. Four patients received WT1 in Montanide, and three with WT1 in poly ICLC. Five patients were in morphologic remission and two had residual morphologic disease at the study entry.

**Results:**

All patients finished the induction phase without any major toxicity except mild transient local injection reaction. One patient on the Montanide arm developed aseptic ulceration at two vaccine sites which healed without antibiotics. Three of 4 patients on the Montanide arm had a decreased expression of WT1 after WT1 vaccination, and two of them demonstrated generation of WT1-specific cytotoxic CD8^+^ T cell responses with biased TCR beta chain enrichment. In contrast, no obvious WT1-specific immune responses were detected in two patients on the poly ICLC arm, nor was there clonal enrichment by TCR alpha/beta sequencing; however, these patients did also have decreased WT1 expression and remained in remission several years after the initiation of treatment.

**Conclusions:**

WT1 peptide vaccine with Montanide as an adjuvant induces detectable WT1-specific CD8^+^ T cell responses with clonal TCR enrichment, which may be capable of controlling leukemia recurrence in the setting of minimal residual disease. Poly ICLC may induce anti-leukemic activity in the absence of detectable WT1 specific CD8^+^ T cell responses.

*Trial registration* NCT01842139, 7/3/2012 retrospectively registered; https://clinicaltrials.gov/ct2/show/NCT01842139.

## Background

While the majority of adults with acute myeloid leukemia (AML) younger than 60 years of age can achieve remission with intensive induction chemotherapy, durable remissions remain elusive. Standard options for eradicating residual leukemia after induction are intensive cycles of cytarabine-based consolidation chemotherapy, autologous stem cell transplant, or allogeneic stem cell transplant. Each option carries significant morbidity and may be difficult to tolerate in patients with co-morbidities and impaired performance status. In the setting of minimal residual disease, immunotherapy offers an innovative strategy for treating post-remission AML without the classic side effects of intensive chemotherapy or transplantation. The existence of leukemia-associated antigens (LAAs), which can serve as molecular markers to distinguish leukemia cells from host cells, provide the rationale for immunotherapy in the treatment of AML [1]. CD8^+^ cytolytic T lymphocytes (CTLs) are capable of recognizing LAAs after presentation on class I MHC molecules, and can subsequently mediate elimination of malignant cells bearing these antigens (reviewed in [2]). Vaccination with peptide antigens can increase the frequencies with which antigen-specific effector and memory T cells are generated to enhance the effectiveness of cancer cell clearance.

Wilms tumor 1 (WT1) is a particularly promising target for cancer vaccination because it is overexpressed in the majority of myeloid cancers and has an essential role in leukemogenesis [3]. The inhibition of *WT1* gene expression causes leukemia suppression in vitro [4, 5] while forced expression of *WT1* in mice results in leukemia induction [6]. In addition, not only do WTI transcript levels in bone marrow and peripheral blood increase with disease severity [7], but detection of WT1 transcripts is itself a marker for minimal residual disease that can presage clinical signs of relapse [8]. These properties make peptide vaccination with WT1 a promising strategy for pursuit in AML.

Two WT1 peptides, the HLA-A*02-restricted peptide 126–134 and the HLA-A*24-restricted peptide 235–243, have been tested in clinical trials and have been shown to be well-tolerated, safe and capable of inducing immunogenic and molecular responses both in the setting of complete remissions (CR) and in active AML and high-risk MDS [9–11]. Vaccination with WT1 peptides can induce expansion of WT1-specific T cells, as measured by tetramer analysis or ELISPOT assay [10–13]. Furthermore, the presence of WT1-specific T cells has been correlated with reduction of WT1 transcript levels (reviewed in [14]).

Given these positive results, the ideal strategy for vaccination with WT1, including identification of the optimal vaccine adjuvant, deserves further investigation. We present the results of a pilot trial of HLA-A02^+^ patients randomized to receive vaccination with WT1 126–134 peptide (RMFPNAPYL) in Montanide or in polyinosinic–polycytidylic acid (poly ICLC), a novel immune adjuvant that acts as a synthetic agonist to Toll-like Receptor 3 (TLR3). In animal models, TLR3 agonists promote an IFN-mediated vaccine response when stimulated by its ligand, dsRNA. dsRNA promotes antigen cross-presentation by dendritic cells and enhances the sustained production of primary and memory CD8^+^ T cell responses [15]. In humans, TLR3 agonists have been used as adjuvant treatments for cancer patients with variable success [16, 17]. The goals of this trial were to compare the effectiveness of a novel immune adjuvant in patients with myeloid leukemia as well as to offer further validation of the immunogenicity of the WT1 vaccine.

## Methods

### Patient selection

Between March 2012 and May 2014, seven adult patients with bone marrow biopsy-confirmed AML or MDS were enrolled. Inclusion criteria were age ≥ 18 years of age, Karnofsky performance status index greater than or equal to 80%, ability to provide written informed consent, adequate hematopoietic, renal and hepatic function, and HLA-A02 expression. Patients were excluded from the study if they were pregnant or nursing, underwent chemotherapy less than 4 weeks prior to the first WT1 vaccine administration, had HIV or other serious concurrent infections including active TB, hepatitis B, or hepatitis C, used concurrent steroids or other immunosuppressive drugs, or had active or a confirmed history of autoimmune disease. Human subjects’ ≤ 18 years were excluded since the WT1 vaccine has not been tested in children. Patients with history of allo-SCT must be off immunosuppression prior to WT1 vaccination. The stem cell donors need to have HLA A0201 to be eligible for the study.

This study conformed to the Declaration of Helsinki and was approved by University of Chicago institutional review board and the FDA IND application. All the participants signed the informed consent before the treatment; and all the participants were consented to allow us to report the clinical research results.

### Study design and treatment

**Fig. 1:**
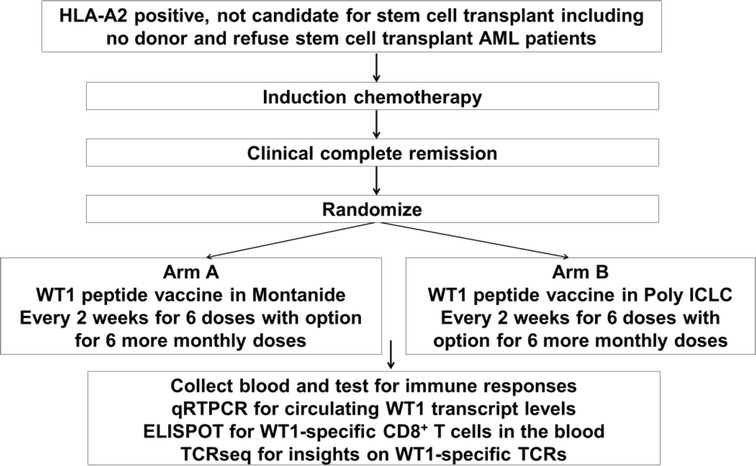
Clinical trial schema

This is an open-label, randomized pilot study assessing administration of WT1 vaccine with Montanide or a TLR3 agonist (poly ICLC) in patients with AML or MDS who were not candidates for stem cell transplant. Patients were screened for WT1 and HLA-A02 expression using pre-induction chemotherapy bone marrow or peripheral blood cells if available. Patients carrying HLA-A02 were randomized to the study within 4 weeks of confirmed CR or CRi. Sealed envelopes were prepared in advance and distributed sequentially to each patient to assign treatment allocation. Patients were randomized to receive 100 μl (1000 mcg) of HLA-A*02-restricted WT1 126–134 peptide (RMFPNAPYL, Multiple Peptide Systems, San Diego, CA) emulsified in either Montanide (Seppic, Inc) or poly ICLC (Oncovir Inc, Washington, DC). 1 mg in 1 ml of aqueous solution was administered subcutaneously every 2 weeks for a total of six injections. Bone marrow biopsies and heparinized blood were collected every 6 weeks to monitor disease progression and molecular and peptide-specific immunological responses. Patients without disease progression after six vaccinations were eligible to receive an additional 6 monthly vaccinations (Fig. 1).

Patients continued in the study until disease relapse, intercurrent illness preventing further treatment, unacceptable adverse events, patient withdrawal from the study, or at the investigator’s discretion. Toxicities were graded according to the National Cancer Institute Common Toxicity Criteria Scale (version 4.0). While an initial sample size of 12 was planned per treatment arm, the study was discontinued once financial support was depleted.

### Gene expression analysis-WT1/ABL

Quantitative real-time PCR (qRT-PCR) was employed to assess the presence of minimal residual leukemia using transcript-specific primer and probe sets for WT1 and ABL genes (20 μM each primer: WT1 5′-CGGTCCGACCACCTGAAG/3′-TTCATCTGACCGGGCAAACT; ABL 5′-AAAATGACCCCAACCTTTTCG/3′-CCATTCCCCATTGTGATTATAGC (IDT Inc). 5 μM WT1 probe: 6FAM-CAGGTAAAACAAGTGAAAAGCCCTTCAGCTGT-TAMRA and 10 μM ABL: 6FAM-TCTAAGCATAACTAAAGGTGAAAAGCTCCGGGTCTT-TAMRA (Biosearch Technology, Inc). Patient bone marrow and/or peripheral blood mononuclear cell cDNA samples synthesized from 5 μg RNA (RNAStat60, Teltest Inc) and no template controls were assayed in triplicate using the LightCycler 480II (Roche). All transcript expression levels were determined by reference to standard curves generated from fivefold serial dilutions of K562 cell line cDNA (0.08–250 ng). The absolute transcript copy number was normalized to the endogenous control gene, *ABL1*.

### WT1 vaccine preparation

Wilms tumor 1 peptide (MPS-173; RMFPNAPYL; PolyPeptide Laboratories, San Diego, CA) vaccines were prepared in the University of Chicago HIM-cGMP facility. Briefly, one vial of WT1 peptide was thawed and mixed with 0.9 ml of sterile water. The diluted WT1 peptide was then either emulsified with Montanide ISA-51 (Seppic, Inc) or mixed with poly-ICLC (Hiltonol, Oncovir) at a 1:1 ratio for injection. The final vaccine product contained 1 mg of WT1 peptide.

### Collection of peripheral blood mononuclear cells (PBMCs) and preparation of CD8^+^ T cells

Heparinized blood was drawn before treatment, monthly during the vaccination period, and at the end of study. Samples were collected prior to a given treatment administration. PBMCs were isolated using Lymphoprep gradient centrifugation and cryopreserved for immune assays. WT1-specific CD8^+^ CTL responses were enumerated by IFN-γ ELISPOT. Low frequency WT1-specific CD8^+^ CTL responses were detected by a direct ex vivo assay, so a short in vitro expansion was performed. Briefly, PBMCs were thawed and washed twice with PBS. CD8^+^ T cells were isolated using anti-CD8 micro beads (Miltenyi Biotech). The non-CD8^+^ population were pulsed with WT1 peptide (50 μM) at the presence of β2-microglobulin for 1 h at 37 °C. The cells were then washed twice with AIM-V media and irradiated at 3000 rad. Purified CD8^+^ T cells were co-cultured with irradiated peptide-pulsed CD8^−^ cells along with IL-2 (10 U/ml) for 5 days. On day 5, the CD8^+^ T cells were collected and re-stimulated with freshly prepared irradiated peptide-pulsed CD8^−^ cells and IL-2 (10 U/ml) for another 5 days. On day 10, the expanded CD8^+^ T cells were collected, counted, and re-stimulated with peptide-loaded T2 cells for IFN-γ ELISPOT analysis.

### ELISPOT assay

Briefly, 96 well multiscreen filter plates were prepared by coating overnight with anti-INF-γ mAb (10 μg/ml), washing 3× with PBS, and blocking 1 h with AIM-V medium containing 10% human AB serum. Expanded CD8^+^ T cells (10,000/well) were added along with T2 cells (50,000/well) previously loaded with WT1 peptide (50 μM). Following a 20 h culture, wells were washed 3 times with ELISPOT wash buffer, incubated 2 h with a biotinylated anti-IFN-γ secondary Ab, washed 3 times, incubated 1 h with streptavidin-conjugated AP, washed, and incubated with AP substrate. Excess substrate was removed by rinsing with tap water. Plates were captured and counted using a CTL-ImmunoSpot S6 Core Analyzer from Cellular Technology Ltd (Cleveland, OH). Stimulation with an irrelevant peptide G250 was used as a negative control, and stimulation with PMA + Ionomycin was used as a positive control for the integrity of the T cell samples. All samples were analyzed in triplicate.

### T cell receptor (TCR) α and β-chain deep sequencing

TCR α- and β-chain deep sequencing was performed to assess clonal enrichment of CD8^+^ T cells throughout vaccination as planned (prior to WT1 vaccination, after 3, 6, 9, and 12 vaccinations if the patient could get) using methodology that has been described previously [18]. The TCR sequencing was done using peripheral blood MNC. In brief, we performed PCR-based amplification of TCRA or TCRB gene products with adapter-conjugated primer sets. The PCR primers were designed to amplify all possible TCRA and TCRB gene products from the V–(D)–J recombination. A forward primer was for the SMART adaptor at 5′-end and a reverse primer was for the constant region of TCRA or TCRB gene, as described previously [18]. The template library was amplified by Nextera XT DNA sample prep kit (Illumina). Subsequently, the prepared library was analyzed using MiSeq Reagent 600-cycle kit v3 and MiSeq system (Illumina). After the deep sequencing, each V, (D), J and C segments in the TCRA and TCRB reference sequences were assigned by determination of amino acid sequences of complement determining region 3 (CDR3) as previously described [18]. The diversity index (inverse Simpson’s index) in CDR3 sequences was calculated to assess overall diversity and clonality in the TCR clonotypes.

## Results

### Patient characteristics and treatment courses

The trial intended to randomize 24 patients to each arm (Fig. 1). Due to difficult to identify HLA A02* patients, and more importantly due to lack of the further funding, the trial was stopped after seven patients were randomized and treated. Between March 2012 and May 2014, seven patients (four males, three female ages 39–73) were randomized as listed in Table 1. Four patients received WT1 in Montanide (three AML, one CML myeloid blast phase, two patients were status post allo-SCT), and three patients received WT1 in poly ICLC (two AML, one MDS RAEB2 status post allo-SCT). Five patients were in morphologic remission (3 in CR1) and two had residual morphologic disease in the marrow at the study entry. All seven patients completed six WT1 vaccines given every 2 weeks. One patient in Montanide group was able to finish all 12 WT1 vaccinations (6 every 2 weeks followed by 6 monthly injection). All the AML patients received 7 + 3 like regimen as the initial induction chemotherapy.

**Table 1:** The clinical characteristics of patients enrolled on the study

Patient no	Diagnosis	Arm	Prior treatments	Disease status at vaccination	WT1 treatment	Treatment after WT1	Severe toxicities	Current status	PFS (days)
1	CML myeloid blast phase	WT1 in Montanide	Failed ponatinib, had MRD SCT with active disease	Morphologic residual disease with molecule disease	s/p 6 WT1 vaccination, tolerated well	DLI 40 days after last WT1 vaccination	No	Died from infection with GVHD	463
2 HLA A0202	Relapsed AML with normal karyotype	WT1 in poly ICLC	CR after induction, 2 cycle HiDAC consolidation, relapse 7 months later, CR2 with clinical trial	Morphologic residual disease, with cytogenetic changes	s/p 6 WT1 vaccination, tolerated well	Disease progressed, and died from disease	Admitted for viral infection (not related)	Died due to disease progression	186
3	t-AML, t (8,21), del 9q	WT1 in poly ICLC	CR1 after induction and consolidation	Morphologic and cytogenetic remission	s/p 9 WT1 vaccination; tolerated well	Off study due to FDA hold of the study	No	In remission	1731
4	MDS RAEB2	WT1 in poly ICLC	CR after MUD SCT	Morphologic and cytogenetic remission	s/p 9 WT1 vaccination; tolerated well	Off study due to FDA hold of the study	No	In remission	1675
5	Relapsed AML after allo-SCT	WT1 in Montanide	CR after 2rd haplo-cord SCT	Morphologic and cytogenetic remission	s/p 7 WT1 vaccination; tolerated well	Off study with neurologic complication	Myelitis after bacterial meningitis	Died in remission	239
6	AML with normal karyotype	WT1 in Montanide	CR after induction/1 cycle consolidation,	Morphologic remission but cytogenetic revolution with 17p deletion	s/p 12 WT1 vaccine	Disease relapsed 18 months after vaccine, treated with decitabine	Developed aseptic ulcer at vaccine sites of 12, 11, 10, and erythema at the 1st vaccine site	Relapsed, Died from disease progression	832
7	AML with complex karyotype	WT1 in Montanide	CR1 after induction, 3 cycle consolidation	Morphologic and cytogenetic remission	s/p 8 WT1, tolerated well, but had disease relapse	Disease progressed after 4 cycle 10 day decitabine	No	Died from disease progression	169

### Toxicities

All patients finished the induction phase without any major toxicity except mild transient local injection reaction (Table 2). One patient (Pt 005) post allo-SCT on the Montanide arm developed transverse myelitis with evidence of bacterial meningitis following the first monthly booster vaccination, which was deemed not to be related to WT1 vaccination. Due to the severity of the event, the study was put on clinical hold by FDA; further vaccination was stopped in the enrolled patients (Pt 003, and Pt 004) who were still receiving treatment. The FDA clinical hold was lifted after the thorough evaluation of the event and two more patients were able to be treated on the study with available funding. Another patient on the Montanide arm developed aseptic ulceration at the 11th and 12th vaccine sites and persistent erythema at the 1st induction vaccine site about 4 weeks after the completion of all 12 WT1 vaccinations. The aseptic ulcers eventually healed with wound care without antibiotics. There were several SAEs not related to WT1 vaccination listed in Table 2.

**Table 2:** Summary of adverse effects on the trial

Adverse events	Total, n (%)	Grade 1 and 2	Grade 3	Grade 4	Grade 5
Related to WT1 vaccine (all with Montanide)					
Injection sites ulceration	1 (14%)		1		
Injection sites reaction	4 (57%)	4			
SAEs unrelated to WT1 vaccine					
Ascending myelitis due to infection	1 (14%)				1
Hyperglycemia due to uncontrolled DM	1 (14%)		1		
Thrombocytopenia due to disease	1 (14%)		1		
Anemia due to disease	1 (14%)		1		
Depression	1 (14%)			1	
Upper respiratory infection (viral)	3 (43%)	3			

### Efficacy

**Fig. 2:**
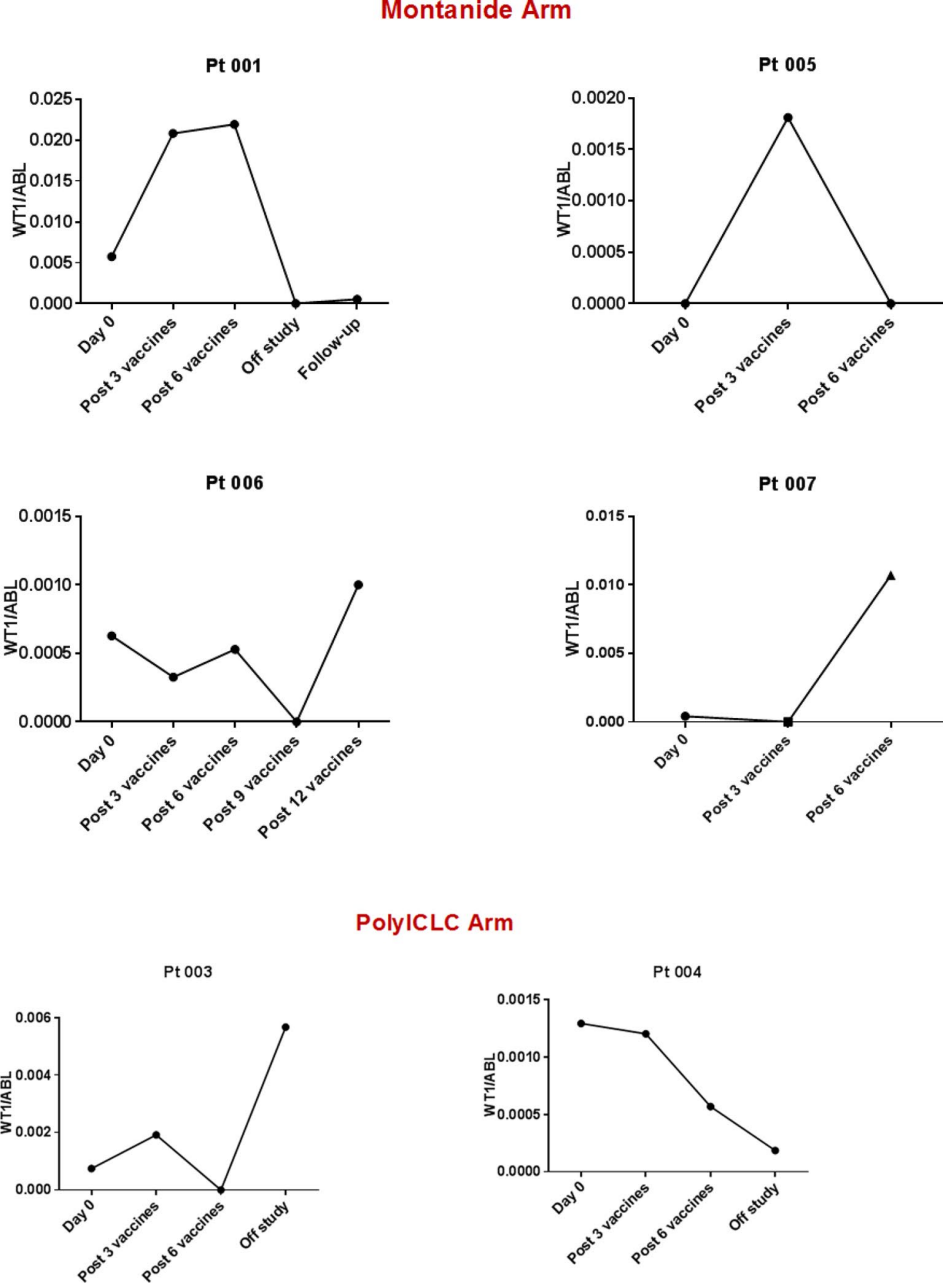
Both WT1 vaccine in Montanide and poly ICLC could decrease WT1 level during the vaccination; and WT1 level is correlated with disease progression

**Fig. 3:**
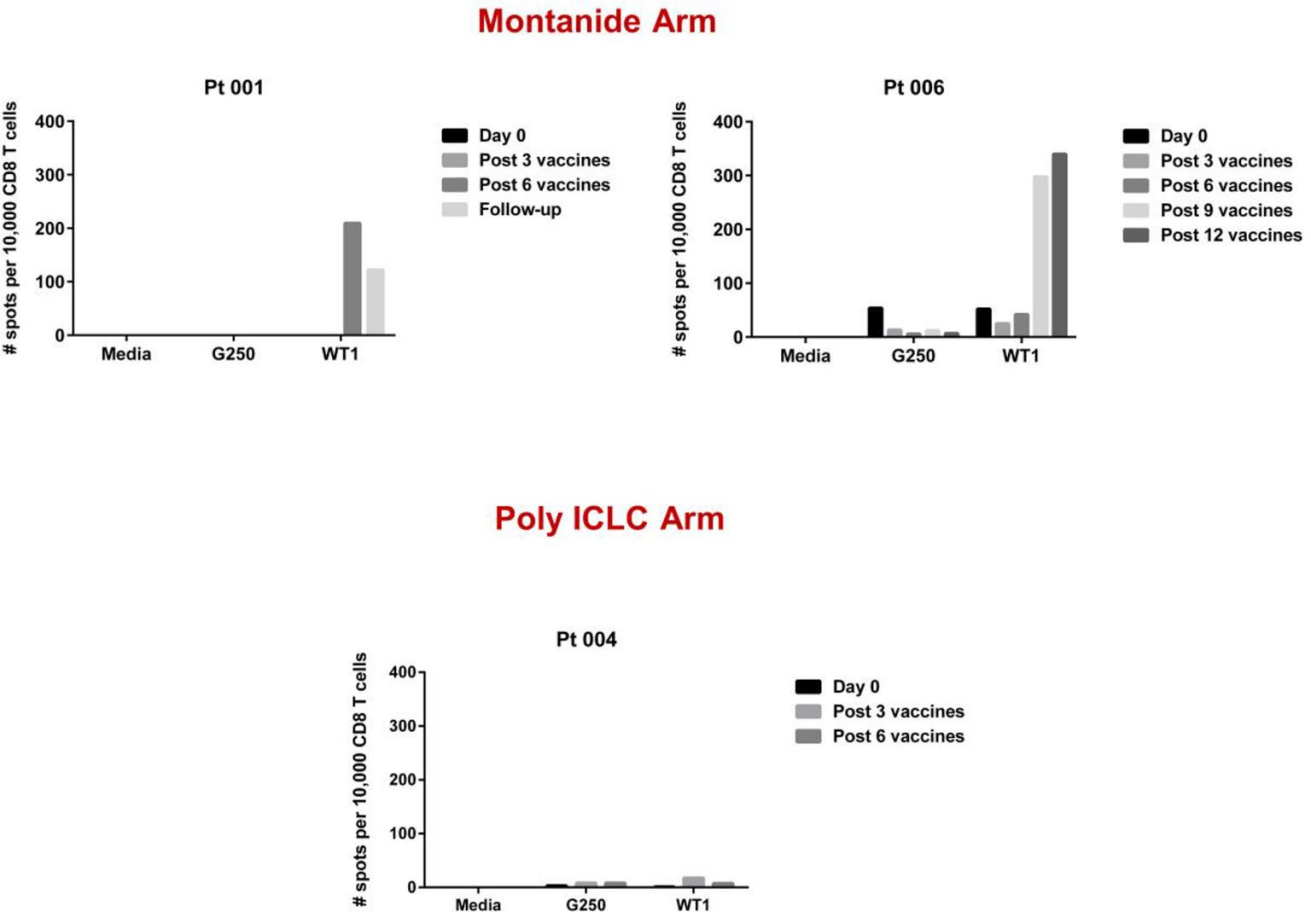
WT1-specific CD8 T cells responses were detected in two patients on the Montanide arm by ELISPOT but not on the poly ICLC arm

Two of 4 patients (Pt 001, Pt 006) on the Montanide arm had deceased expression of WT1 transcripts (assessed by qRT-PCR) after WT1 vaccination (Fig. 2) associated with generation of WT1-specific cytotoxic CD8^+^ T cell responses (Fig. 3). Both had delayed WT1 qRT-PCR responses: Pt 001 had increased WT1 PCR level after the third and sixth WT1 vaccination, thus donor lymphocyte infusion (DLI) was planned. Interestingly, the WT1 PCR level became undetectable at the time prior to DLI about 40 days after 6th WT1 vaccination, and remained very low when last measured at the 3 month follow-up (Fig. 2, Pt 001) with persistent WT1-specific CD8^+^ T cells (Fig. 3, Pt 001). The WT1 PCR level of Pt 006 was undetectable after 9 WT1 vaccines, but unfortunately resurged after 12 vaccinations. This patient subsequently relapsed several months later (Fig. 2, Pt 006). Both Pt 001 and Pt 006 had increased WT1 level at the time of disease relapse. WT1 specific CD8^+^ T cells were detected with WT1 peptide vaccination (Fig. 3, Pt 006). The high level WT1 specific CD8^+^ T cell at the time of WT1 PCR relapse might help to control the leukemia, since the patient did not have morphologic relapse until 18 months after the last WT1 vaccination. Pt 005 had some increase of WT1 PCR level after three vaccinations, but it became undetectable after the 6th WT1 vaccination. Patient 007 had a mild decrease of WT1 level after the three vaccinations, but it increased sharply following the 6th WT1 vaccination, corresponding to morphologic disease relapse. Our data from this small number of patients suggest that monitoring WT1 levels during vaccination treatment might be useful to predict response and disease relapse.

In comparison to the Montanide group, WT1 peptide in poly ICLC resulted in decrease of WT1 PCR levels especially in Pt 004 (Fig. 2, Pt 004). However, WT1 peptide in poly ICLC failed to induce any detectable WT1 specific CD8^+^ lymphocytes (Fig. 3, Pt 004 and data not shown). Thus, our data from the limited patients demonstrated that Montanide appears to be a superior vaccine adjuvant to poly ICLC for induction of specific CD8^+^ T cell responses. However, poly ICLC might lead to control of WT1 levels through other mechanisms. Our observation will need to be confirmed by a large study.

### Biased TCR enrichment with WT1 peptide vaccination

**Fig. 4:**
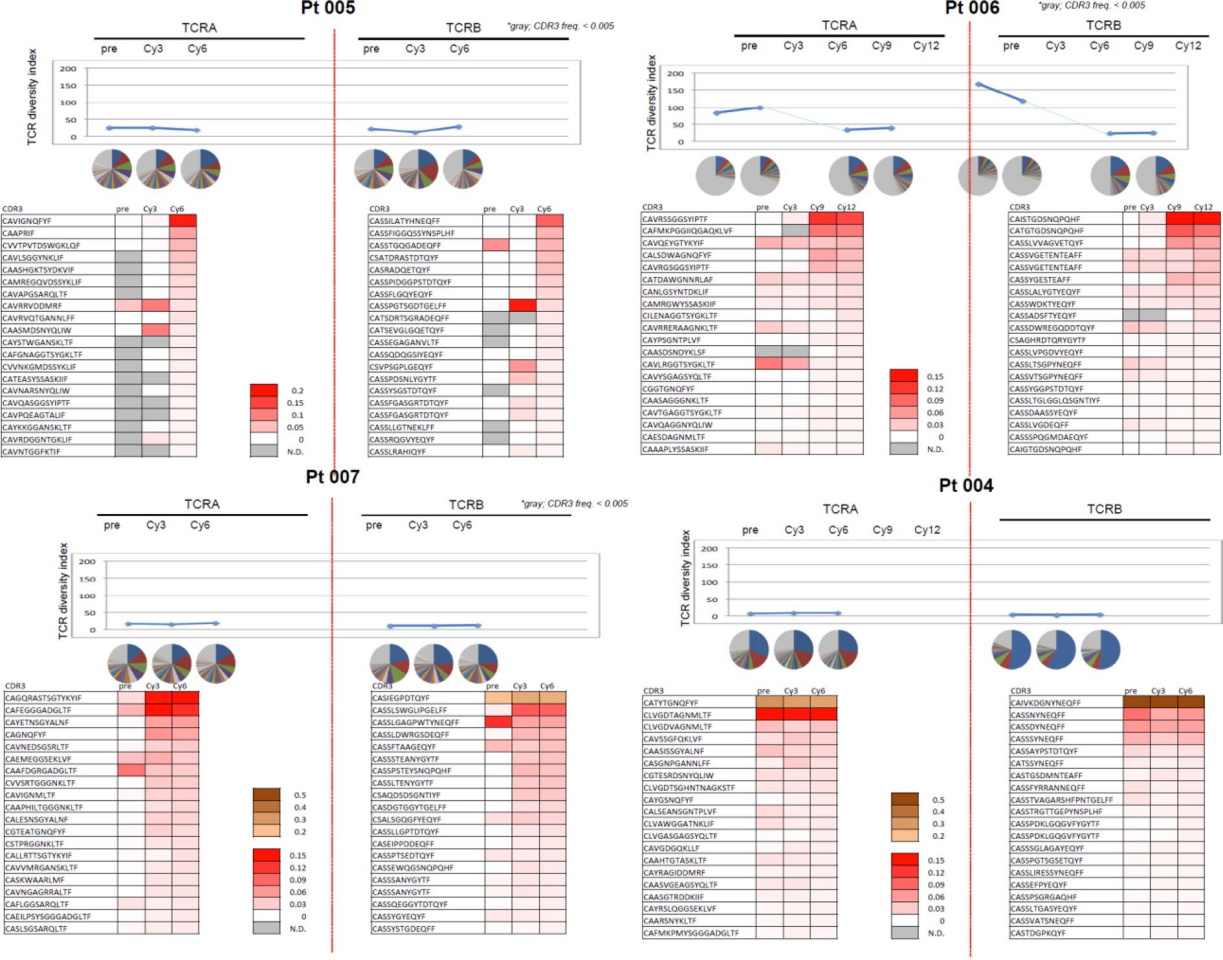
Clonal enrichment of CD8 T cells was detected in three patients on Montanide arm (Pt 005, Pt 006 and Pt 007) by TCR deep sequencing but not in the patient (Pt 004) on the poly ICLC arm. For Pt 006, Pt 007, and Pt 004; TCR sequencing was done prior to vaccination, after 3 and 6 cycle vaccination; for Pt 006, TCR sequencing was done prior to vaccination, after 3, 6, 9 and 12 vaccination

In order to monitor the TCR repertoire changes with WT1 vaccination, TCR alpha and beta deep sequencing was done on three patients on the Montanide arm and one patient on the poly ICLC arm from whom cells were available for analysis. As shown in Fig. 4, Pt 005, Pt 006 and Pt 007 on the WT1 in Montanide arm showed biased enrichment of CDR3 clonotypes, indicating clonal expansion of certain CD8^+^ T cell populations. In contrast, in the two patients on the poly ICLC arm from whom no specific CD8^+^ T cell response was detected, no clonal expansion was observed by TCR alpha/beta sequencing (Pt 004 was shown in Fig. 4). The third patient on the poly ICLC arm was later found to have A0202 instead of A0201, and thus would not have been expected to respond and could serve as negative control. Not surprisingly, this patient did not have a decrease of WT1 qRT-PCR levels nor TCR clonal expansion during vaccination (data not shown). The patient tolerated the vaccine well without injection reactions and had stable AML for 12 weeks, but the disease progressed before the first monthly WT1 (7th) vaccination.

## Discussion

Our pilot study was initially designed to determine the better adjuvant (Montanide vs poly ICLC as a TLR3 agonist) for vaccination against the HLA-A2-binding peptide from the WT1 antigen. To our knowledge, this is the first prospective study to directly compare two adjuvants using WT1 peptide vaccination with incorporation of WT1 minimal residual disease monitoring and TCR repertoire analysis. Although we could not complete the whole enrollment of the trial, our data suggest that Montanide may be the superior adjuvant to induce peptide specific cytotoxic T cells and could be considered for future vaccine studies in patients. Due to the small number of patients randomized to each arm, statistical analysis could not applied; we are reporting our interesting observation without statistical significance in the hope to provide some insight for the future trial design.

Our trial demonstrated that it is plausible to incorporate minimal residual disease monitoring and TCR repertoire analysis in prospective studies with coordinated efforts. This helped us to understand the different mechanisms that Montanide and poly ICLC as adjuvants might use to control disease. We anticipated generation of WT1 specific CD8^+^ CTL after WT1 peptide in Montanide vaccination, which resulted in transient control of disease elicited by the WT1 MRD monitoring; these results are consistent with previous results from other groups [11, 14, 19, 20]. An interesting finding from our study was that administration of WT1 in the TLR3 agonist poly ICLC was associated with clinical benefit manifested by controlling of WT1 PCR levels, and two patients remained in clinical remission more than 3 years after treatment (we could not rule out that their remission might not associated with WT1 vaccination in poly ICLC), even though no increase in WT1-specific CD8^+^ T cell response was detected. It is possible that, given the small number of patients treated; these results are a consequence of variability in the WT1 assay over time. However, it is conceivable that poly ICLC stimulates immune activation and disease control through different mechanisms, distinct from induction of CD8^+^ T cells. When engaged, TLRs promote the priming of adaptive immune responses by host antigen presenting cells [21]. TLR signaling on dendritic cells following CpG or LPS exposure has been reported to render effector T cells refractory to Treg-mediated suppression [22]. TLR3 agonists have been utilized to treat cancer patients with the aim of inducing an IFN-mediated anticancer immune response [17]. Poly-ICLC has been given intramuscularly or subcutaneously and has been used safely in several clinical trials with promising efficacies in various type of tumors [23, 24]. Poly ICLC was demonstrated to induce rapid immune response in ovarian cancer patients when used as an adjuvant in targeting tumor self-antigens [25]. Rapoport et al. [26] demonstrated that combination of Montanide/poly ICLC/MAGE-A3 protein had greater antibody responses and better CTL responses in patients with multiple myeloma but was complicated with severe injection-site reactions that evolved into sterile abscess, but the MAGE-A3 alone without Montanide group still elicited clinical responses. Due to the small number of patients on each arm, our observations were informative only and solid conclusion will require larger study.

Recent advances in deep sequencing technology make it possible to characterize the antigen-specific T cell receptor repertoire generated following immunotherapies in cancer patients (i.e. the “clonality” of the response). Previous studies demonstrated biased usage of TCR-Vβ gene families in WT1 peptide-vaccinated patients [27, 28]. Thus, an in-depth analysis of the WT1 specific TCR repertoire could lead to identification of high avidity WT1-specific TCRs for use in future adoptive cell therapy approaches [29–31]. Our TCR sequencing results demonstrated TCR clonal enrichment with vaccination of WT1 in Montanide, but not in WT1 in poly ICLC. These results suggest that different mechanisms are involved in the apparent disease control by WT1 peptide vaccination in these two adjuvants. With the accumulation of novel WT1-specific TCR sequences from vaccinated patients, it may be possible to establish a dataset or bank of WT1-specific TCR sequences to be used as a pool to generate WT1 reactive TCR engineered CD8^+^ T cells for clinical utilization as cellular therapy.

Our results demonstrated efficacy and tolerability of WT1 peptide vaccination in patients with myeloid malignancies especially in the setting of minimal residual disease status. In order to enhance the efficacy of peptide vaccination, future work should include blockade of negative regulatory mechanisms such as depletion of Tregs, or the inclusion of checkpoint inhibitors such as anti-CTLA4 antibody or anti-PD1/PD-L1 antibodies.

## Abbreviations

WT1: Wilms tumor 1
TLR3: Toll-like receptor 3
poly ICLC: polyinosinic–polycytidylic acid
qRT-PCR: quantitative real-time PCR
CDR3: complement determining region 3
AE: adverse event
AML: acute myeloid leukemia
allo-SCT: allogeneic stem cell transplantation
DLI: donor lymphocyte infusion
